# Supplementary material for: Biomechanics of the peafowl’s crest reveals frequencies tuned to social displays
Source: PLoS One. 2018 Nov 28;13(11):e0207247. doi: 10.1371/journal.pone.0207247 (PMC6261573; doi:10.1371/journal.pone.0207247)
Supplement: S1 Table — These values are provided for comparison with data shown in Fig 3 for peafowl crests. (PDF) [file pone.0207247.s002.pdf]

S1 Table. Individual feathers used in the vibrational resonant measurements. These values are provided for comparison with data shown in Fig. 3 for peafowl crests.

Species	Feather type	Rachis length (cm) (+/- 0.05 cm 95% CI)
Indian peafowl male	Body contour semiplume	3.94
Indian peafowl male	Body contour semiplume	5.95
Indian peafowl male	Wing covert	10.67
Indian peafowl male	mantle	5.15
Indian peafowl male	mantle	7.00
Indian peafowl male	train eyespot	11.50
Victoria crowned pigeon	crest	6.30
Victoria crowned pigeon	crest	12.50
Himalayan monal	crest	5.50
Golden pheasant	crest	5.00
Yellow-crested cockatoo	crest	10.00
