# Supplementary material for: Biomechanics of the peafowl’s crest reveals frequencies tuned to social displays
Source: PLoS One. 2018 Nov 28;13(11):e0207247. doi: 10.1371/journal.pone.0207247 (PMC6261573; doi:10.1371/journal.pone.0207247)
Supplement: S2 Table — (PDF) [file pone.0207247.s003.pdf]

S2 Table. Sound pressure levels (SPL) of avian wingbeats during flight and wing-beating displays measured in previous studies.

Bird species	SPL (dB)	Distance (m)	Reference
Eastern phoebes, *Sayornis phoebe*	64-66 dB SPL at 1 kHz	1.2 m	Fournier, J.P., Dawson, J.W., Mikhail, A., & Yack, J.E. (2013). If a bird flies in the forest, does an insect hear it? Biology letters 9(5): 20130319.
Black-capped chickadees, *Poecile atricapillus*	54-60 dB SPL at 25 kHz	1.2 m	Fournier, J.P., Dawson, J.W., Mikhail, A., & Yack, J.E. (2013). If a bird flies in the forest, does an insect hear it? Biology letters 9(5): 20130319.
Crested pigeons, *Ocyphaps lophotes*	≤ 67.6 dB SPL	1.0 m	Hingee, M. & Magrath, R.D. (2009) Flights of fear: a mechanical wing whistle sounds the alarm in a flocking bird. Proceedings of the Royal Society of London B: rspb20091110.
Ruffed grouse, *Bonasa umbellus* (a wing-beating display)	66.2 dB SPL, bandwidth 300 Hz to 8 kHz, frequency weighting not reported	1.0 m	Garcia, M., Charrier, I., & Iwaniuk, A.N. (2012) Directionality of the drumming display of the ruffed grouse. The Condor 114(3): 500-506.
