# Supplementary material for: Biomechanics of the peafowl’s crest reveals frequencies tuned to social displays
Source: PLoS One. 2018 Nov 28;13(11):e0207247. doi: 10.1371/journal.pone.0207247 (PMC6261573; doi:10.1371/journal.pone.0207247)
Supplement: S3 Table — (PDF) [file pone.0207247.s004.pdf]

S3 Table. Wingflap frequencies of adult peacocks during level and ascending flight.

Source	Number of individuals	Wingflap frequency (Hz)
https://www.youtube.com/watch?v=HvY_1wFSFsQ accessed September 28, 2017	1	4.92
https://www.youtube.com/watch?v=7gxQwm4MWns accessed September 28, 2017	1	4.16
https://www.youtube.com/watch?v=U55iMIiI_k0 accessed September 28, 2017	1	6.43
https://www.youtube.com/watch?v=kZe0jLkeMuk accessed September 28, 2017	1	4.42
https://www.youtube.com/watch?v=FrMQs7OwWC8 accessed September 28, 2017	3	6.36<br>6.00<br>5.55
https://www.youtube.com/watch?v=A5xSgaXDkTY accessed September 28, 2017	2	5.81<br>6.15
