# Supplementary material for: Biomechanics of the peafowl’s crest reveals frequencies tuned to social displays
Source: PLoS One. 2018 Nov 28;13(11):e0207247. doi: 10.1371/journal.pone.0207247 (PMC6261573; doi:10.1371/journal.pone.0207247)
Supplement: S4 Table — (PDF) [file pone.0207247.s005.pdf]

**S4 Table. Symbols used to indicate different crest samples in Figures 2 and 3 of the main text.**

| Symbol | Sample ID | Sex |
|---|---|---|
| ○ | Crest 07 | female (peahen) |
| △ | Crest 08 | female |
| + | Crest 10 | female |
| × | Crest 11 | female |
| ◇ | Crest 12 | female |
| ▽ | Crest 13 | female |
| ⊠ | Crest 14 | female |
| ✳ | Crest 15 | female |
| ○ | Crest 01 | male (peacock) |
| △ | Crest 02 | male |
| + | Crest 03 | male |
| × | Crest 04 | male |
| ◇ | Crest 05 | male |
| ▽ | Crest 06 | male |
| ⊠ | Crest 09 | male |
