# Supplementary material for: Biomechanics of the peafowl’s crest reveals frequencies tuned to social displays
Source: PLoS One. 2018 Nov 28;13(11):e0207247. doi: 10.1371/journal.pone.0207247 (PMC6261573; doi:10.1371/journal.pone.0207247)
Supplement: S5 Table — (PDF) [file pone.0207247.s006.pdf]

S5 Table. Best-fit models of f_r and Q in the analysis of the vibrational dynamics measurements.

Response	Fixed-effect	Estimate (SE)	t	p
f_r	Orientation (in-plane vs. out-of-plane)	–2.22 (0.23)	–9.46	< 0.0001
	Sex (male vs. female)	0.26 (1.49)	0.18	0.86
	Top area	–0.82 (0.46)	–1.78	0.10
Q	Orientation (in-plane vs. out-of-plane)	–1.26 (0.25)	–4.94	< 0.0001
	Sex (male vs. female)	1.85 (0.49)	3.71	0.005
	Top area	–0.37 (0.15)	–2.40	0.04
