# Supplementary material for: Biomechanics of the peafowl’s crest reveals frequencies tuned to social displays
Source: PLoS One. 2018 Nov 28;13(11):e0207247. doi: 10.1371/journal.pone.0207247 (PMC6261573; doi:10.1371/journal.pone.0207247)
Supplement: S6 Table — There are many bird species wherein both sexes have a flexible feather crest. To understand the taxonomic breadth of birds that have shaking displays in addition to the crest, we used natural history resources including photos, videos and descriptive accounts of appearance and behavior. We documented at least 35 species across 10 different orders in which the females exhibit flexible feather crests and the males are known to perform shaking displays. (PDF) [file pone.0207247.s007.pdf]

S6 Table. Species in which both sexes have crests of flexible feathers and the male also performs a shaking display. There are many bird species wherein both sexes have a flexible feather crest. To understand the taxonomic breadth of birds that have shaking displays in addition to the crest, we used natural history resources including photos, videos and descriptive accounts of appearance and behavior. We documented at least 35 species across 10 different orders in which the females exhibit flexible feather crests and the males are known to perform shaking displays.

Order	Species	Common name
Accipitriformes	*Sagittarius serpentarius*	Secretary bird
Cariamiformes	*Cariama cristata*	Crested cariama
Columbiformes	*Geophaps plumifera*	Spinifex pigeon
	Goura cristata	Western crowned pigeon
	Goura scheepmakeri	Southern crowned pigeon
	Goura victoria	Victoria crowned pigeon
	Ocyphaps lophotes	Crested pigeon
Galliformes	*Afropavo congensis*	Congo peafowl
	Argusianus argus	Great argus
	Colinus cristatus	Crested bobwhite
	Leipoa ocellata	Malleefowl
	Lophophorus impejanus	Himalayan monal
	Lophura ignita	Crested fireback
	Lophura leucomelanos	Kalij pheasant
	Pavo cristatus	Indian peafowl
	Pavo muticus	Green peafowl
	Polyplectron bicalcaratum	Gray peacock pheasant
	Polyplectron malacense	Malayan peacock pheasant
	Polyplectron napoleonis	Palawan peacock pheasant
	Polyplectron schleiermacheri	Bornean peacock pheasant
	Rheinardia ocellata	Crested argus
	Tetrao urogallus	Western capercaillie
Gruiformes	*Balearica pavonina*	Black crowned crane
	Balearica regulorum	Gray crowned crane
Opisthocomiformes	*Opisthocomus hoazin*	Hoatzin
Passeriformes	*Baeolophus bicolor*	Tufted titmouse
	Cardinalis cardinalis	Northern cardinal
	Onychorhynchus coronatus	Royal flycatcher
	Prionops plumatus	White-crested helmetshrike
	Pycnonotus jocosus	Red-whiskered bulbul
	Rupicola peruvianus	Andean cock-of-the-rock
Pelicaniformes	*Phalacrocorax auritus*	Double-crested cormorant
	Phalacrocorax carbo	Great cormorant
Suliformes	*Anhinga anhinga*	Anhinga
Tinamiformes	*Eudromia elegans*	Elegant crested tinamou
