# Supplementary material for: Biomechanics of the peafowl’s crest reveals frequencies tuned to social displays
Source: PLoS One. 2018 Nov 28;13(11):e0207247. doi: 10.1371/journal.pone.0207247 (PMC6261573; doi:10.1371/journal.pone.0207247)
Supplement: S1 Fig — (A) Apparatus for measuring the vibrational response of peafowl feather crests. Crests were first glued onto balsa wood blocks, which were then mounted on a mechanical shaker driven by a function generator that produced a sine wave output with a linear ramp in the frequency of shaking. The resulting motions of the crest flags and the shaker were measured using high-speed video. (B) Feather samples from S1 Table measured for comparison with peafowl crest vibrational resonant frequencies (not shown to scale): (i), (ii) peacock mantle feathers; (iii) short peacock eyespot feather; (iv), (v) peafowl body semiplumes; (vi) peacock wing covert; (vii) Himalayan monal crest feather); (viii) yellow crested cockatoo crest feather; (ix), (x) Victoria crowned pigeon crest feathers; (xi) golden pheasant crest feather. (PDF) [file pone.0207247.s008.pdf]

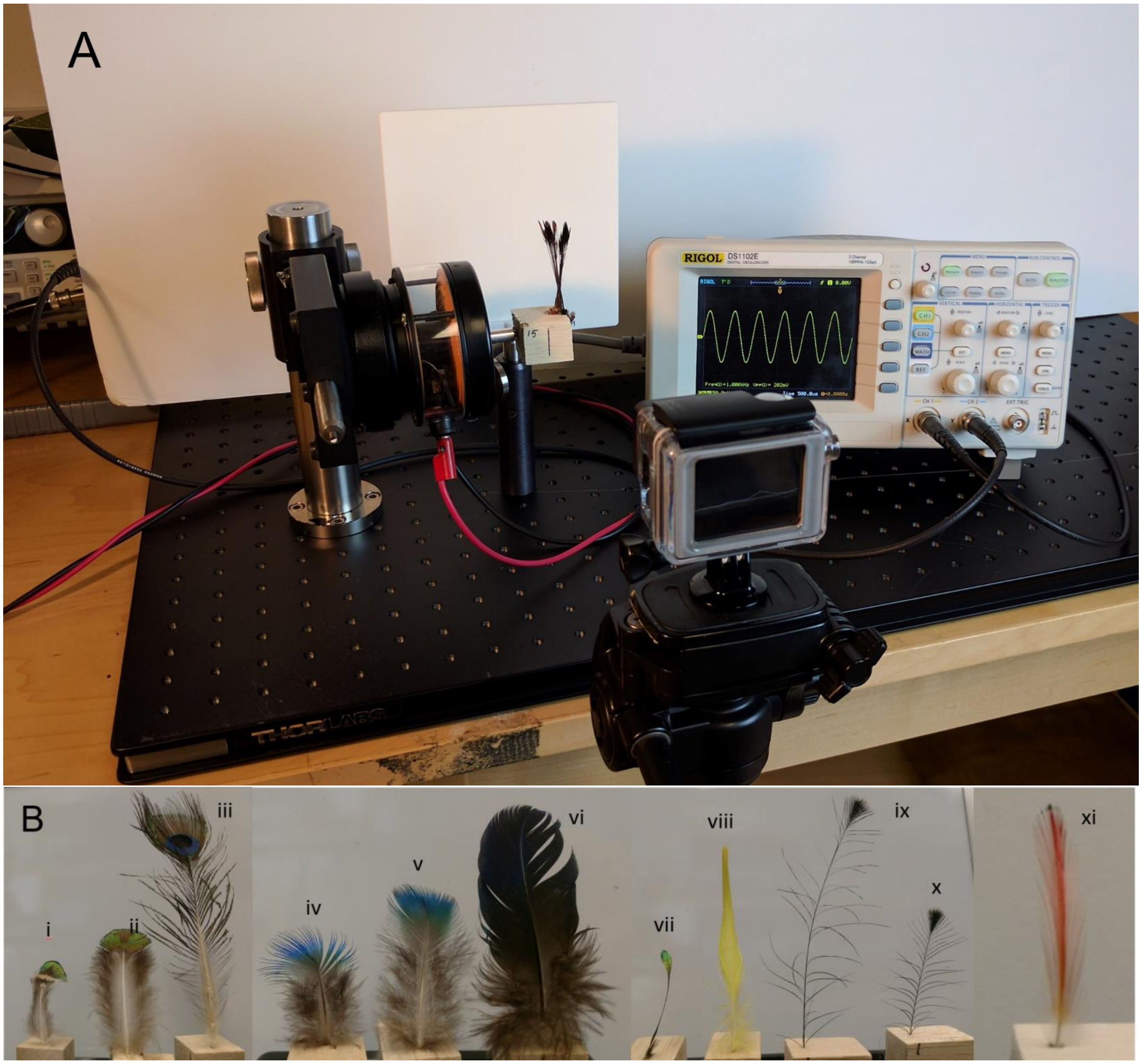

S1 Fig. Vibrational response apparatus and feather samples. (A) Apparatus for measuring the vibrational response of peafowl feather crests. Crests were first glued onto balsa wood blocks, which were then mounted on a mechanical shaker driven by a function generator that produced a sine wave output with a linear ramp in the frequency of shaking. The resulting motions of the crest flags and the shaker were measured using high-speed video. (B) Feather samples from Table S1 measured for comparison with peafowl crest vibrational resonant frequencies (not shown to scale): (i), (ii) peacock mantle feathers; (iii) short peacock eyespot feather; (iv), (v) peafowl body semiplumes; (vi) peacock wing covert; (vii) Himalayan monal crest feather); (viii) yellow crested cockatoo crest feather; (ix), (x) Victoria crowned pigeon crest feathers; (xi) golden pheasant crest feather.
