# Supplementary material for: Biomechanics of the peafowl’s crest reveals frequencies tuned to social displays
Source: PLoS One. 2018 Nov 28;13(11):e0207247. doi: 10.1371/journal.pone.0207247 (PMC6261573; doi:10.1371/journal.pone.0207247)
Supplement: S2 Fig — (A-B) An example waveform and spectrogram for one of the playback stimulus tracks of peacock train-rattling sounds. The waveform (A) and spectrogram (B) were generated from a re-recording made of the playback stimulus, to ensure that features of the playback stimulus matched those of the original recording from Dakin et al. (2016). The white box in (B) highlights a single rattle note in the train-rattling spectrogram. (C) Playback apparatus viewed from above. The crest sample (red ellipse) was exposed to the flow near-field of a loudspeaker (top) that played back peacock train-rattling sounds. The resulting motions of the crest flags were measured using a high-speed video camera (located to the right of the ellipse). (D-E) Waveform and spectrogram of the white noise control played back through the same audio system used for train-rattling playbacks. This illustrates the resemblance between the broad-band frequency spectrum of the white noise control (E) and the rattle notes (B). However, the white noise control lacks modulation at the low frequencies characteristic of displays. (PDF) [file pone.0207247.s009.pdf]

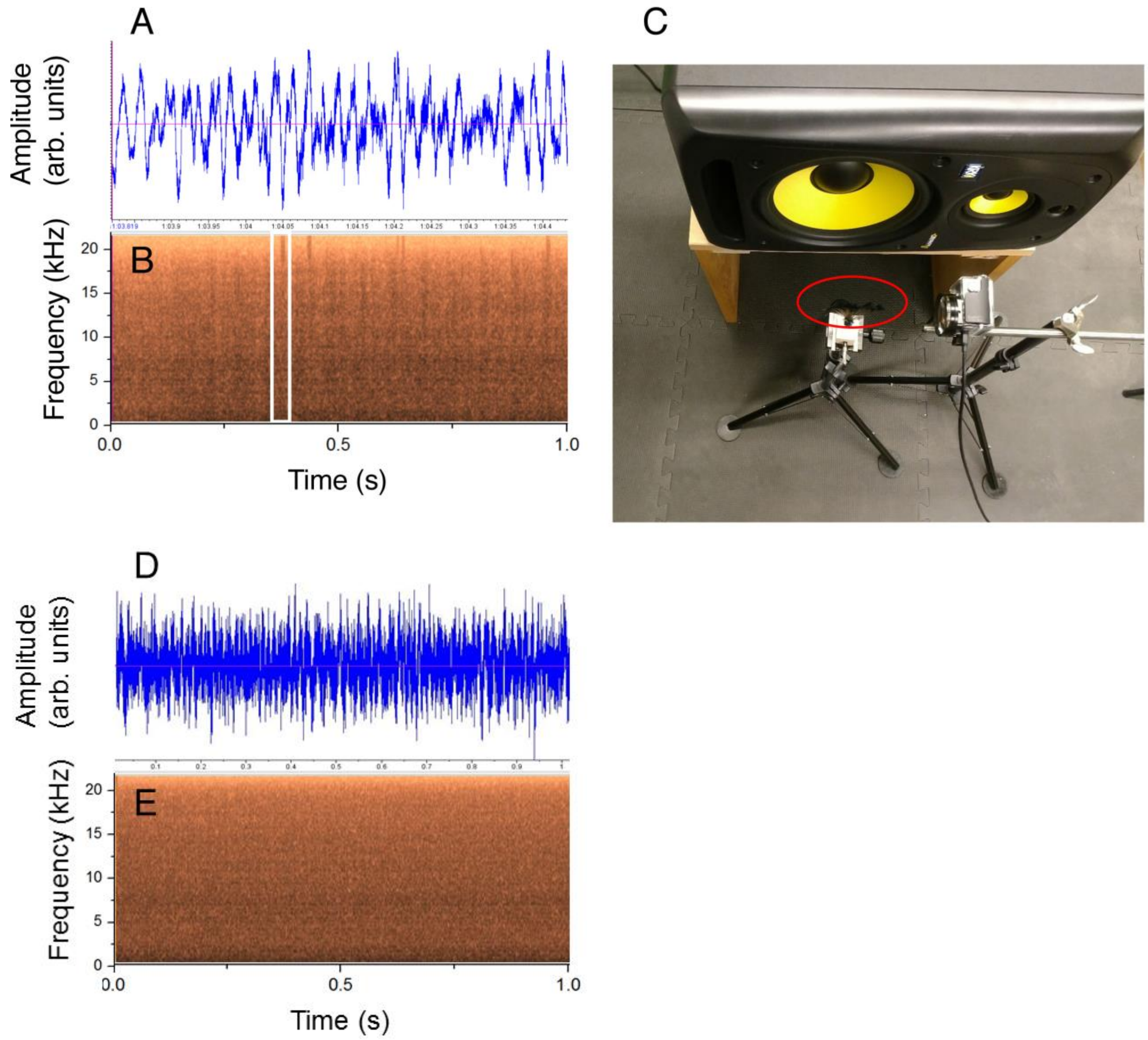


**S2 Fig. Example audio and apparatus used for measuring the vibrational response of peafowl feather crests during audio playback of peacock train-rattling mechanical sounds.** (A-B) An example waveform and spectrogram for one of the playback stimulus tracks of peacock train-rattling sounds. The waveform (A) and spectrogram (B) were generated from a re-recording made of the playback stimulus, to ensure that features of the playback stimulus matched those of the original recording from Dakin et al. (2016). The white box in (B) highlights a single rattle note in the train-rattling spectrogram. (C) Playback apparatus viewed from above. The crest sample (red ellipse) was exposed to the flow near-field of a loudspeaker (top) that played back peacock train-rattling sounds. The resulting motions of the crest flags were measured using a high-speed video camera (located to the right of the ellipse). (D-E) Waveform and spectrogram of the white noise control played back through the same audio system used for train-rattling playbacks. This illustrates the resemblance between the broad-band frequency spectrum of the white noise control (E) and the rattle notes (B). However, the white noise control lacks modulation at the low frequencies characteristic of displays.
