# Supplementary material for: Biomechanics of the peafowl’s crest reveals frequencies tuned to social displays
Source: PLoS One. 2018 Nov 28;13(11):e0207247. doi: 10.1371/journal.pone.0207247 (PMC6261573; doi:10.1371/journal.pone.0207247)
Supplement: S3 Fig — Peacock wing-shaking displays were simulated using a peacock wing mounted on a carbon fiber rod. The rod was rotated at approximately 5 Hz (a typical wing-shaking frequency) about a clevis joint located at the wing’s shoulder joint, ensuring that the plane of the wing’s surface remained vertical while the tips circumscribed a 14 cm diameter circle. Peahen crests were positioned in the region of maximum airflow at distances ≤ 90 cm (50 cm shown here) from the wingtips. The resulting crest motion was filmed using high-speed video. (PDF) [file pone.0207247.s010.pdf]

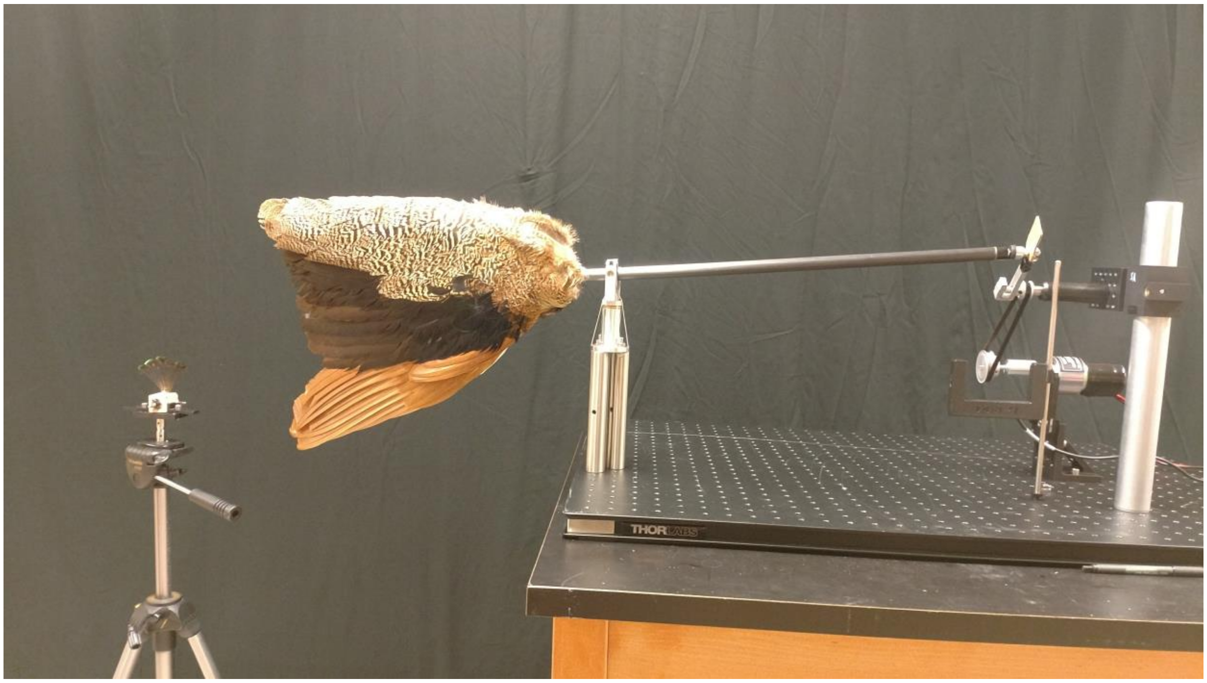

S3 Fig. Robotic apparatus for simulating peacock wing-shaking. Peacock wing-shaking displays were simulated using a peacock wing mounted on a carbon fiber rod. The rod was rotated at approximately 5 Hz (a typical wing-shaking frequency) about a clevis joint located at the wing's shoulder joint, ensuring that the plane of the wing's surface remained vertical while the tips circumscribed a 14 cm diameter circle. Peahen crests were positioned in the region of maximum airflow at distances ≤ 90 cm (50 cm shown here) from the wingtips. The resulting crest motion was filmed using high-speed video.
