# Supplementary material for: Biomechanics of the peafowl’s crest reveals frequencies tuned to social displays
Source: PLoS One. 2018 Nov 28;13(11):e0207247. doi: 10.1371/journal.pone.0207247 (PMC6261573; doi:10.1371/journal.pone.0207247)
Supplement: S4 Fig — Force-displacement trials were performed three times each for n = 3 male and n = 3 female crests, respectively. The bending spring constant, k, was calculated from the slope of linear model fits to the resulting force-displacement data from each trial. The example in (A) shows data from a single trial on peacock Crest 09 to illustrate the linearity of the response, with symbols scaled to span y-axis measurement error. (B) Values of k from each of three trials on the total n = 6 crests. Each crest sample is denoted by a different symbol-color combination, following Figs 2 and 3 of the main text, and ordered left to right by decreasing mean k value. Blue data are male (peacock) crests and green data are female (peahen) crests. (PDF) [file pone.0207247.s011.pdf]

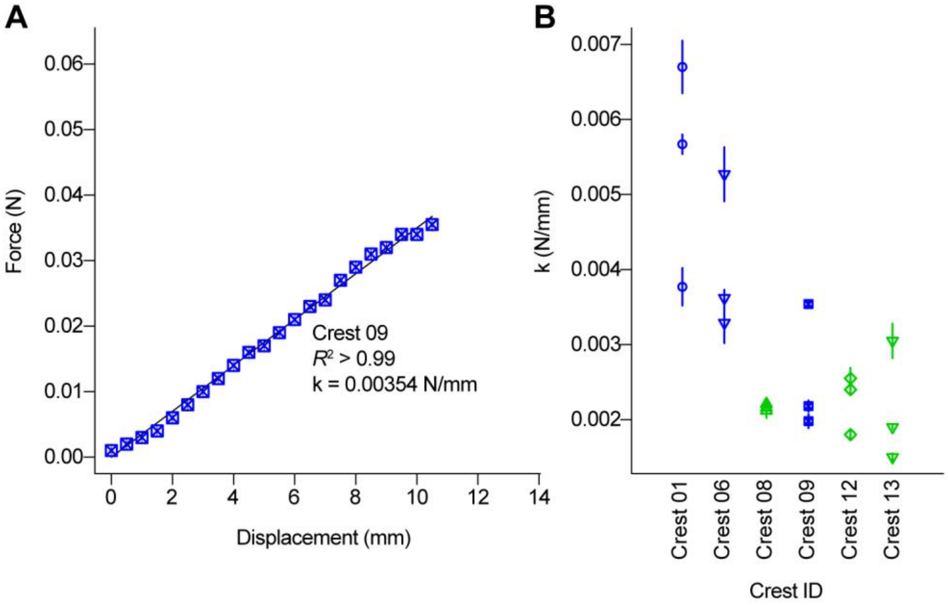

S4 Fig. Bending spring constant, k, of peafowl crests. Force-displacement trials were performed three times each for n = 3 male and n = 3 female crests, respectively. The bending spring constant, k, was calculated from the slope of linear model fits to the resulting force-displacement data from each trial. The example in (A) shows data from a single trial on peacock Crest 09 to illustrate the linearity of the response, with symbols scaled to span y-axis measurement error. (B) Values of k from each of three trials on the total n = 6 crests. Each crest sample is denoted by a different symbol-color combination, following Figs. 2-3 of the main text, and ordered left to right by decreasing mean k value. Blue data are male (peacock) crests and green data are female (peahen) crests.
